# Recognition of EEG Features in Autism Disorder Using SWT and Fisher Linear Discriminant Analysis

**DOI:** 10.3390/diagnostics15182291

**Published:** 2025-09-10

**Authors:** Fahmi Fahmi, Melinda Melinda, Prima Dewi Purnamasari, Elizar Elizar, Aufa Rafiki

**Affiliations:** 1 Department of Electrical Engineering, Universitas Sumatera Utara, Medan 20155, Indonesia; 2 Department of Electrical and Computer Engineering, Universitas Syiah Kuala, Banda Aceh 23111, Indonesia; melinda@usk.ac.id (M.M.); elizar.mustafa@usk.ac.id (E.E.); aufarafi21@mhs.usk.ac.id (A.R.); 3 Department of Electrical Engineering, Universitas Indonesia, Depok 16424, Indonesia; prima.dp@ui.ac.id

**Keywords:** Autism Spectrum Disorder (ASD), electroencephalogram (EEG), Fisher Linear Discriminant Analysis (FLDA), Stationary Wavelet Transform (SWT), confusion matrix

## Abstract

**Background/Objectives**: An ASD diagnosis from EEG is challenging due to non-stationary, low-SNR signals and small cohorts. We propose a compact, interpretable pipeline that pairs a shift-invariant Stationary Wavelet Transform (SWT) with Fisher’s Linear Discriminant (FLDA) as a supervised projection method, delivering band-level insight and subject-wise evaluation suitable for resource-constrained clinics. **Methods**: EEG from the KAU dataset (eight ASD, eight controls; 256 Hz) was decomposed with SWT (db4). We retained levels 3, 4, and 6 (γ/β/θ) as features. FLDA learned a low-dimensional discriminant subspace, followed by a linear decision rule. Evaluation was conducted using a subject-wise 70/30 split (no subject overlap) with accuracy, precision, recall, F1, and confusion matrices. **Results**: The β band (Level 4) achieved the best performance (accuracy/precision/recall/F1 = 0.95), followed by γ (0.92) and θ (0.85). Despite partial overlap in FLDA scores, the projection maximized between-class separation relative to within-class variance, yielding robust linear decisions. **Conclusions**: Unlike earlier FLDA-only pipelines and wavelet–entropy–ANN approaches, our study (1) employs SWT (undecimated, shift-invariant) rather than DWT to stabilize sub-band features on short resting segments, (2) uses FLDA as a supervised projection to mitigate small-sample covariance pathologies before classification, (3) provides band-specific discriminative insight (β > γ/θ) under a subject-wise protocol, and (4) targets low-compute deployment. These choices yield a reproducible baseline with competitive accuracy and clear clinical interpretability. Future work will benchmark kernel/regularized discriminants and lightweight deep models as cohort size and compute permit.

## 1. Introduction

Neurodevelopmental Autism Spectrum Disorder (ASD) is a complex cranial nerve disorder that significantly impacts the growth and behavioral development of affected children [1]. This syndrome is characterized by repetitive behavioral patterns [2]. Based on research, many children with autism remain undiagnosed or do not receive appropriate treatment, largely due to environmental factors and the inadequacy of diagnostic equipment [3]. With advances in technology, especially in the use of an electroencephalogram (EEG) [4,5], detecting autism in children is potentially becoming easier. This can be achieved by recording brain signals via a Brain–Computer Interface (BCI) with a collection of electrodes placed on the scalp [4]. However, with complex EEG signals, this research requires a Machine Learning (ML) scheme to analyze signal patterns that are difficult to analyze. The ML technique effectively utilizes real-time processing methods for EEG signals [5]. Automation of the detection method reduces reliance on manual inspection by highly specialized experts, leading to quicker responses and potentially enhancing the overall outcomes of classification management [6].

The integration of ML into biomedical fields is not only revolutionizing diagnostics but also optimizing operations across various industries, including manufacturing, where it has proven effective in cost estimation [7]. The potential of ML to enhance the accuracy of autism predictions is significant, potentially saving time and resources in clinical settings [8].

Recent studies have demonstrated the effectiveness of ML in detecting brain abnormalities through EEG. For instance, Shi et al. established a predictive model differentiating between autism and neurotypical development, achieving an accuracy of 85.4% using k-Nearest Neighbors (kNN) pipeline on the KAU dataset [9]. Further advancements by Aljalal et al. employed Variational Mode Decomposition (VMD) in conjunction with kNN to detect Mild Cognitive Impairment (MCI), achieving a remarkable accuracy of 99.81% [10]. Similarly, Al-Jumaili et al. demonstrated the utility of various ML algorithms with feature extraction methods for detecting epileptic seizures, obtaining an accuracy of 96% with Support Vector Machine (SVM) [11]. In the context of autism detection, Melinda et al. utilized SVM combined with Continuous Wavelet Transform (CWT) to achieve a 95% classification accuracy for autistic children [12]. Chaddad et al. provided a comprehensive analysis of various EEG signal classification techniques, underscoring the potential of ML algorithms to achieve high accuracy [13].

Among the various ML methods, Fisher Linear Discriminant Analysis (FLDA) is particularly notable for its application in statistics and pattern recognition. This technique effectively classifies data while maximizing class separation, making it an ideal choice for our study [14,15]. This method was chosen because it can produce good accuracy and improve system performance [16,17]. FLDA is a supervised learning technique that is used to reduce dimensions while classifying data by maximizing the distance between classes [18]. We extend the prior DWT + LDA pipeline by adopting SWT and FLDA to address two known limitations. First, SWT (undecimated) is shift-invariant, mitigating the translation-variance and down-sampling artifacts of DWT and yielding more stable EEG sub-band features for short, non-stationary segments [13]. Second, we employ FLDA as a supervised projection (≤C−1 components) rather than the LDA classifier, to reduce dimensionality and avoid small-sample covariance instability typical in high-dimensional EEG [19]. This method is combined with the Stationary Wavelet Transform (SWT) method to filter and extract signals at certain frequencies. This research builds upon a previous journal article that utilized Discrete Wavelet Transform (DWT) extraction and Linear Discriminant Analysis (LDA) in processing EEG data from individuals with autism and those without [16]. The difference lies in the component extraction method used, which utilizes only one detailed component, represented by a beta signal. The results of this follow-up research include the classification of three-component extractions and the accuracy of implementing FLDA on autistic and normal EEG signals.

Stationary Wavelet Transform (SWT) is a digital signal processing technique that simultaneously filters and extracts signals to certain frequencies through Low-Pass Filter (LPF) and High-Pass Filter (HPF) processes [20]. SWT extraction will produce three attributes in the form of levels 3, 4, and 6 components, which are gamma, beta, and theta signals, respectively. This component is a feature chosen to test the performance of the FLDA system in classifying signals [13]. The use of this wavelet technique can also help increase noise suppression in EEG signals through denoising techniques. The advantage of using the SWT technique is that it does not require down-sampling or up-sampling. In this case, the number of coefficients and diffusion levels remains constant, or the length of the transformation signal at each level is fixed [21].

Research on ASD EEG is increasingly adopting deep-learning architectures and advanced time–frequency/connectivity pipelines [13], with representative ASD studies using spectro-temporal features and engineered classifiers reporting strong performance [12,22,23]. In this context, we position our SWT–FLDA approach as a compact, interpretable baseline evaluated subject-wise, which complements modern models by (i) reducing overfitting risk in small cohorts, (ii) providing band-level discriminative insight (β vs. γ/θ), and (iii) aligning with resource-constrained clinical deployment.

The objective of this study is to develop a robust classification system for EEG signals from autistic and typically developing children by combining FLDA with SWT. This approach aims to enhance signal extraction and noise suppression, thereby facilitating more accurate classification of ASD and typical EEG data. The study’s major contributions can be summarized as follows. This research presents a novel approach that integrates Fisher Linear Discriminant Analysis (FLDA) with the Stationary Wavelet Transform (SWT) for classifying EEG signals from autistic and normal children. The study offers an in-depth evaluation of the system’s performance based on confusion matrix metrics, including accuracy, precision, recall, and F1-score. A comparative analysis of FLDA’s effectiveness in classifying multiple EEG components (gamma, beta, and theta signals) extracted through SWT highlights the method’s versatility.

This research aims to enhance clinical diagnostics and improve early intervention strategies for children with autism by providing a reliable and automated detection method for ASD through EEG analysis. Our SWT–FLDA pipeline is lightweight and interpretable, suitable for resource-constrained clinics (e.g., in Indonesia) where routine deployment of large deep-learning models is impractical.

## 2. Materials and Methods

**Figure 1:**
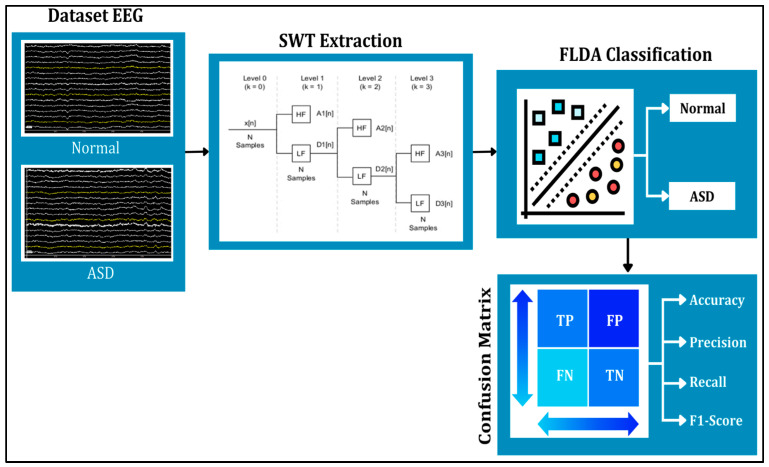
The proposed framework, different colors illustrates normal and ASD classification.

This study applied the SWT and FLDA methods to process the data (see Figure 1). SWT was used to denoise signals and extract signals into several different frequencies. The results of SWT extraction are presented in the form of three frequency attributes, corresponding to components at levels 3, 4, and 6, which represent gamma, beta, and theta signals, respectively. The denoising or noise suppression process in SWT utilized the thresholding feature. The three levels of filtering and decomposition results were later used as features for FLDA classification testing. Meanwhile, FLDA was used to classify signals in autistic and normal syndromes. This research aims to compare the accuracy results of EEG classification for autistic and normal individuals using the FLDA technique, based on head condition in terms of frequencies at each level.

### 2.1. Datasets

This study used an EEG dataset provided by King Abdulaziz University (KAU), Jeddah, Saudi Arabia [24]. This dataset comprises 16 EEG signals, including 8 EEG samples from autistic children and 8 EEG samples from typically developing children, recorded using the BCI2000 viewer in a .dat format with a 16 × 16 (trial × channel) structure. Each sample contains 16 channels consisting of FP1, F3, F7, T3, T5, O1, C4, FP2, FZ, F4, F8, C3, CZ, PZ, OZ, and O2, following the international 10–20 system. We retained the 16-channel 10–20 montage (Fp1…O2) to bilaterally sample regions implicated in ASD (frontal, temporal, central, parietal, occipital), given prior ASD EEG reports of reduced/atypical frontal and fronto-posterior coherence and altered spectral power (decreased alpha with increases in theta/beta, with reports of elevated high-frequency activity), which motivates this coverage [25,26,27].

The dataset ensures the confidentiality of all participants by excluding any personal identification information; ASD subtypes are not annotated in the publicly available description, and the annotations consist of group labels (ASD vs. control) rather than per-event clinical markers. The recorded EEG signals represent a diverse demographic, including eight subjects with ASD (five males and three females, aged 6 to 20 years; total recorded duration of 4104.2 s) and eight control subjects (all males, aged 9 to 13 years; total recorded duration of 4534.9 s). The KAU Hospital clinical team assigned group labels (ASD vs. control); we used these labels as the ground truth and performed no re-labeling. The public dataset provides group-level annotations only (no per-event clinical markers and no ASD subtypes). The public description does not disclose the specific diagnostic instruments or protocols used to establish these labels (e.g., ADOS/ADI-R, DSM-5), which we note as a limitation of the source data. This inclusion of a range of ages and both genders among the ASD participants increases the validity and generalizability of the research findings. The EEG recordings were captured using Ag/AgCl electrodes in conjunction with a g.tec EEG deviceand a g.tec USB amplifier (g.tec medical engineering GmbH, Schiedlberg, Austria), and BCI2000 (Wadsworth Center, Albany, NY, USA) while the subjects were in a relaxed state. The recordings were digitized at 256 Hz with an acquisition band-pass of 0.1–60 Hz and a 60 Hz notch. For researchers interested in accessing this dataset, inquiries can be directed to Dr. Mohammed Jaffer Alhaddad via email at malhaddad@kau.edu.sa.

Annotations were performed by qualified clinical staff following standard diagnostic procedures for ASD at KAU Hospital, although specific instruments (e.g., ADOS/ADI-R) were not disclosed in the public dataset [24]. As reported in [24], the original EEG data collection at KAU Hospital was conducted under the approval and oversight of the KAU Ethics Committee. Written informed consent was obtained from all participants or their legal guardians prior to data acquisition, and all recordings were fully anonymized before release. The present work involved no new data collection or direct contact with human participants; only secondary analysis of the anonymized dataset was performed.

### 2.2. Data Input Preprocessing

In this stage, EEG datasets were input with a sampling frequency of 256 Hz. At the data import stage, the function type used is BCI2kReader (the BCI2000 file I/O utility), because the EEG signal recording format has an output in BCI2000 “.dat” format and was acquired using the BCI2000 system. BCI2000 is a modular platform consisting of Operator, Source, Signal Processing, and Application modules. In our setup, the gUSBamp Source module interfaced the g.tec USB amplifier, and the native .dat files included a header with acquisition parameters and channel labels. Of these sixteen recorded channels, fifteen were analyzed (Fp1…Oz); channel O2 was excluded from analysis. All subsequent preprocessing, SWT feature extraction, and FLDA classification were performed on this 15-channel matrix. Then, to obtain information from the signal, event information from each recorded channel was imported, namely, 16 events per data sample. This process produced a data length of 16,000 for 15 EEG samples. Then, the data was exported in double (number) form to produce a signal output in .txt data format.

### 2.3. Stationary Wavelet Transform (SWT)

Feature extraction and selection techniques significantly contribute to improving BCI capabilities across various applications, including cognitive enhancement and neuroprosthetics [28]. This research utilized SWT to decompose and filter signals into smaller dimensions through the Low-Pass Filter (LPF) process, which filters low-frequency signals, and the High-Pass Filter (HPF) process, which filters high-frequency signals. SWT is a modified method of Discrete Wavelet Transform (DWT), also known as Undecimate Wavelet Transform. SWT does not include down-sampling or up-sampling, so the number of coefficients and decomposition levels remains constant at each level, resulting in faster computation. The filtering process produces subsample results as described in the following formula [29]:

(1) $$\Psi_{b}^{a}=\frac{1}{\sqrt{a}}\psi \text{*}\left(\frac{t-b}{a}\right),a>0,b\epsilon R$$

where $\psi_{b}^{a}$ is the mother wavelet, a is a scale parameter, b is a shift parameter, and ψ*(t) is the complex conjugate of the mother wavelet. The discrete values and shift parameters are a = 2j and b = 2j k.

SWT utilizes a thresholding function to remove noise by setting small-amplitude wavelet coefficients (representing noise) to zero or a specified threshold value [30]. The choice of threshold value must be optimal. A threshold value that is too small can leave noise, while one that is too large can erase valid signal information. Therefore, selecting an optimal threshold value is crucial to minimize the Mean Squared Error (MSE) [31]. There are two basic wavelet shrinkage functions, namely, hard threshold and soft threshold [32]. We selected SWT because it is undecimated/translation-invariant, yielding shift-robust multi-scale representations and enabling wavelet-shrinkage denoising without training data, which is appropriate for small-sample EEG. Prior work also showed SWT’s advantage over DWT for EEG artifact removal [33,34]. While deep-learning super-resolution methods (e.g., Wang et al., 2023) can enhance time–frequency concentration, they typically require larger datasets and careful tuning; for our small-sample EEG cohort, SWT provided a reproducible, non-learned alternative with lower overfitting risk [35]. We restricted features to SWT levels corresponding to θ (level 6), β (level 4), and γ (level 3) to balance physiological relevance with our small-sample setting and to limit dimensionality (reducing overfitting risk). Recent ASD EEG studies have indicated robust alterations in higher-frequency features (e.g., decreased α and increased β/γ), whereas δ is highly state-dependent and prone to low-frequency/ocular contamination in short 4 s epochs. Accordingly, α/δ coefficients were decomposed but not used as predictive inputs in the present model. We note this as a limitation and a target for future work (e.g., δ/α ratios) [36,37].

### 2.4. Hard Threshold

A hard threshold is a linear function that removes coefficients below a threshold value determined by the noise variance. The hard threshold filter coefficient is obtained using

(2) $$\Delta_{\lambda }^{H}\left(x\right)=\left\{\begin{array}{c} x,\;if\;\left|x\right|>\lambda \;\left(keep\right)\\ 0,\;otherwise\;\left(kill\right) \end{array}\right.$$

This wavelet is known as a “keep or kill” procedure. Thresholding is better known because the thresholding function includes discontinuity; therefore, x values above the lambda threshold are not affected. Conversely, if they are below the lambda threshold, they will be changed to 0.

### 2.5. Soft Threshold

The soft thresholding function is typically used because it remains continuous, coefficients with $\left|d_{j}\left[n\right]\right|>\lambda_{j}$ are shrunk toward zero by $\lambda_{j}$, while those with $\left|d_{j}\left[n\right]\right|<\lambda_{j}$ are set to zero. The soft threshold filter can be stated as follows:

(3) $$\hat{d_{j}}\left[n\right]=\mathrm{sign}\left(d_{j}\left[n\right]\right)\max\left(\left|d_{j}\left[n\right]\right|-\lambda_{j},0\right)$$

where $\hat{d_{j}}\left[n\right]$ denotes the SWT detail coefficient at level j, $\lambda_{j}$ is the level-dependent threshold, and $\hat{d_{j}}\left[n\right]$ is the thresholded coefficient. The primary difference between the hard and soft threshold functions is that the coefficient below the threshold is set to zero in the hard threshold. Thus, this will weaken the signal oscillations, and the reconstructed signal will have poor smoothness [38]. Based on the graph, the signal created will follow the shape of the original signal; however, if the coefficient is less than or equal to the threshold value, it will be set to zero, which can compromise the interpretation of the data. Meanwhile, with soft thresholding, the graph created will follow the shape of the signal without destroying the data. The following is an SWT chart for decomposing and extracting signals into smaller frequencies.

**Figure 2:**
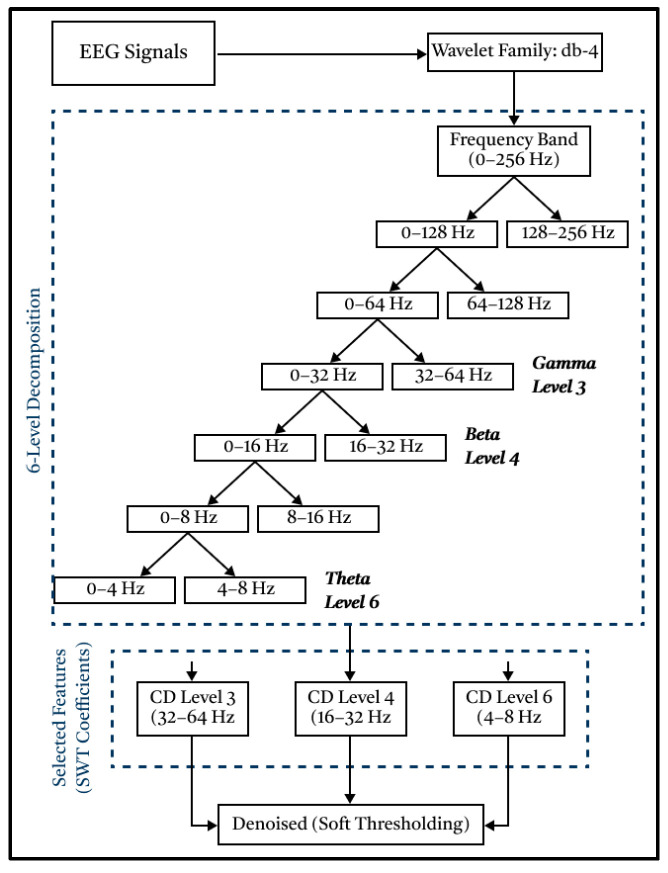
SWT processing method.

Based on Figure 2, the EEG signal will be extracted, decomposed, and filtered into 6 levels through a High-Pass Filter (detail component) and Low-Pass Filter (approximation component) process. The mother wavelet is Daubechies-4 (db4), selected for its compact support, orthogonality, four vanishing moments, and strong time–frequency localization, properties widely leveraged in EEG denoising and sub-band feature extraction [39]. Prior Q1/Q2 works have commonly used db4 for EEG decomposition/rhythm features, and recent analyses emphasized that the mother wavelet substantially influences the informative content of wavelet features. This wavelet has proven to be very suitable for processing raw EEG signals and to produce the best signal filtering [40]. Consistent with these reports, db4 provided stable coefficients for our data and yielded robust filtering performance [39]. Next, the three detailed components produced in the form of signals at levels 3, 4, and 6 were utilized, corresponding to gamma, beta, and theta signals, respectively. These three levels were chosen because they can represent the conditions of autistic and normal children when they are sleeping, active, and focused.

The denoising process was carried out by utilizing threshold and sampling functions through the Low-Pass Filter and High-Pass Filter processes. This type of soft thresholding was chosen to eliminate noise because it can smooth the reconstructed signal without distorting it, thereby preserving the signal integrity [20]. Therefore, the resulting signal graph will retain the same information as the original signal graph.

### 2.6. Fisher Linear Discriminant Analysis (FLDA)

We used Fisher Linear Discriminant Analysis (FLDA), a supervised linear method that learns a projection w, maximizing between-class separation relative to within-class variance. For two classes, the Fisher criterion is

$$J\left(w\right)=\frac{w^{⊤S_{b}w}}{w^{⊤S_{w}w}}$$

where $S_{b}\;$and $S_{w}$ are the between- and within-class scatter matrices; the solution reduces to the generalized eigenproblem $S_{b}v=\lambda S_{w}v$, and classification proceeds with a linear decision boundary in the projected space [41,42,43]. Here, we used FLDA explicitly as a supervised projection (feature extractor). In contrast, the classical LDA classifier models class-conditional Gaussians with a shared, full-rank covariance estimated in the original feature space; under small-sample and correlated features, this pooled covariance can be ill-conditioned/singular, degrading LDA classification. FLDA sidesteps this by learning ≤C−1 discriminant components that maximize $S_{b}/S_{w}$ and then applying a linear rule in the projected space [19].

FLDA projects feature vectors into a direction that pushes class means apart while compressing within-class spread. In binary cases, this yields a one-dimensional representation where classes are maximally separated, and in multi-class cases, up to C−1 discriminant components are obtained [41,42,43]. In practice, features were z-normalized before FLDA to align scales across SWT features. This projection-first design is motivated by the Small-Sample-Size (SSS) regime typical in EEG studies (*n* ≪ d), for which recent work refines LDA/trace-ratio formulations precisely to address SSS pathologies, supporting our choice of FLDA projection for stability.

**Figure 3:**
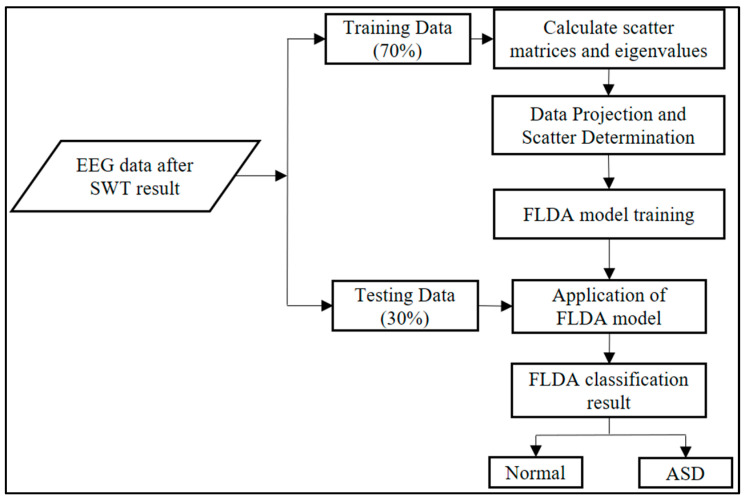
FLDA system performance settings.

In our pipeline, SWT features served as inputs. We used a subject-wise, stratified 70/30 train–test split (Figure 3). Given the small cohort and the absence of hyperparameter tuning (FLDA has a closed-form solution), we did not allocate a separate validation split. All preprocessing (e.g., z-normalization) was fit on the training set only to prevent leakage, and performance on the held-out test set was summarized using accuracy, precision, recall, and F1-score. This follows recommended practice in small-sample biomedical ML (hold-out when no tuning; nested cross-validation when tuning is required) [44,45].

In EEG classification, commonly used models include linear (FLDA/LDA, logistic regression), SVM, k-NN, tree ensembles, and deep networks (CNN/LSTM/graph) [13] In view of our small-sample setting and hand-crafted SWT features, we adopted FLDA as a low-variance, interpretable baseline with minimal hyperparameter tuning and a closed-form solution, which is advantageous for reproducibility [14]. Classical ML remains competitive for ASD EEG when data are limited, as shown in recent open-access studies [8]. Exploration of SVM/ensembles/DL with larger cohorts remains for future work [13].

We intentionally employed a single, closed-form FLDA classifier to obtain a stable, interpretable subject-wise baseline in this small cohort. Broader model families (k-NN/SVM/RF/CNN/LSTM) have been well documented in prior work [8,12,13,14], but direct numerical comparisons are not meaningful here due to different datasets and validation protocols; therefore, we treat them as context rather than benchmarks.

## 3. Results

This section reports results for classifying autism vs. normal EEG using SWT features and FLDA.

### 3.1. Data Input Preprocessing Results

In processing normal and autistic EEG data, only 15 of the 16 active channels were read, namely, FP1, F3, F7, T3, T5, O1, C4, FP2, FZ, F4, F8, C3, CZ, PZ, and OZ. Channel O2 is not read because it is considered a grounding channel. The input process involved 16 iterations for 16 patients with 15 channels, a total of 240,000 features. The dataset comprises 16,000 data points, divided into 8000 normal EEG data points and 8000 autistic EEG data points. Labels on EEG channels indicate electrode positions on the head, such as F (Frontal), Fp (Frontopolar), C (Central), O (Occipital), and T (Temporal).

### 3.2. Stationary Wavelet Transform (SWT) Result

In this study, SWT was used to extract and filter signals to certain frequencies using Daubechies 4 (db-4) as the mother wavelet. Extraction yielded three detailed components, namely, levels 3, 4, and 6, which represent the gamma signal, beta, and theta, respectively. The results obtained from feature extraction and denoising for the normal and ASD classes are presented in Table 1, Table 2 and Table 3 below.

The results of the SWT data in the frequency range of 32–64 Hz (level 3) indicate that the patient’s brain activity is in a focused state (Table 1 and Table 4). The frequency range of 16–32 Hz (level 4) shows normal brain activity (Table 2 and Table 5). The frequency range of 4–8 Hz (level 6) shows brain activity in sleep (Table 3 and Table 6). From the results obtained, the normal EEG is predominantly higher and more stable compared to the autistic EEG. The electrical activity of normal children experiences greater fluctuations, meaning that the channels in the normal EEG are more active. Meanwhile, frequency fluctuations produced by autistic children experience inconsistent up-and-down changes and tend to be smaller. These conditions can affect the accuracy of FLDA classification performance.

### 3.3. Fisher Linear Discriminant Analysis (FLDA) Result

**Figure 4:**
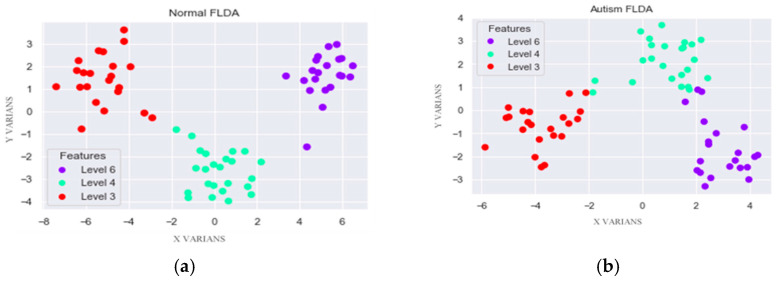
Results of FLDA classifications: (**a**) normal class and (**b**) autism class.

FLDA’s primary goal is to effectively separate different features by transforming data into a lower-dimensional space, maximizing the distance between classes, and minimizing the distance within the same classes. FLDA takes normal and autistic EEG datasets, each of which has 15 active channel parameters, and converts them into one representative value. FLDA creates new axes based on data characteristics, which reduces variance and maximizes the distance between classes. The results of normal and autistic EEG classification show good separation based on each feature, as shown in Figure 4.

Building on this, FLDA projects the SWT-based EEG feature vectors onto a single discriminant axis that maximizes between-class separation (autism vs. normal) while minimizing the within-class variance. Each sample is mapped to a discriminant score and classified in a binary setting with autism as the positive class and normal as the negative class.

Figure 4 visualizes the FLDA outputs by SWT component—level 3 (γ, red), level 4 (β, green), and level 6 (θ, blue). The normal (–) class shows clear separation; autism (+) exhibits some overlap due to non-linear structure, but this does not preclude accurate discrimination, as quantified below.

Table 4 reports the test-set confusion matrices (subject-wise, stratified 70/30 split; *n* = 4800 segments per level; autism = 2400, normal = 2400) with autism as the positive class. Performance is highest for level 4 (β), with accuracy = 0.950 (TP = 2280; TN = 2280), followed by level 3 (γ), with accuracy = 0.920 (TP = 2208; TN = 2208), and level 6 (θ), with accuracy = 0.850 (TP = 2040; TN = 2040). These results confirm that β-band features are the most discriminative on this dataset.

### 3.4. Hyperparameters

Signals were processed as described in Section 2; Stationary Wavelet Transform (SWT) features were extracted at levels 3, 4, and 6. All features were z-normalized (mean 0, unit variance) using statistics computed on the training set only.

We employed Fisher’s Linear Discriminant (FLDA) as a supervised projection; for C classes, up to C−1 discriminant components were retained. A linear decision rule with equal class priors was applied in the projected space. FLDA is closed-form and does not require iterative hyperparameter tuning.

A subject-wise, stratified 70/30 train–test split was used (no subject overlap). Preprocessing transforms were fit on the training set and applied to the test set to avoid leakage. The random seed was fixed (e.g., 42) to ensure reproducibility. No early stopping or model selection was performed.

All experiments were run using standard scientific Python libraries (Python 3.10); code-level settings (e.g., data loaders, batch sizing if applicable) follow defaults, as no iterative optimization was required by FLDA.

### 3.5. Evaluation Criteria

Table 5 summarizes the class-wise metrics computed under the same convention (autism = positive): recall/sensitivity (autism), specificity (normal), precision (autism), F1-score (autism), and accuracy. In line with the confusion matrices, level 4 (β) attains precision = 0.950, recall = 0.950, F1 = 0.950, and accuracy = 0.950; level 3 (γ) yields precision = 0.908, recall = 0.932, F1 = 0.920, and accuracy = 0.920; and level 6 (θ) yields precision = 0.846, recall = 0.854, F1 = 0.850, and accuracy = 0.850.

## 4. Discussion

This study aimed to evaluate the application of the FLDA system in classifying EEG signals from individuals with autism and those without autism, based on accuracy. This classification system separates signals based on detailed components at each level. The results show an accuracy of above 90%, indicating a high level of classification accuracy. FLDA is a suitable choice for ML techniques on autistic and normal EEG signals.

Although the autism and normal score distributions overlap in the FLDA-projected space (Figure 4), linear separability was not required. FLDA chooses a projection that maximizes between-class separation relative to pooled within-class variance, after which a single threshold yields a robust linear decision; this explains the high β-band performance (precision/recall/accuracy = 0.95; Table 4 and Table 5) despite partial overlap. Residual errors near the overlap likely reflect non-linear structure and will be explored with kernel/regularized discriminants under the same subject-wise protocol.

Neurophysiological interpretation: From a neurophysiology perspective, the relatively higher and more stable activity observed in the normal group versus the smaller, more inconsistent fluctuations in the autism group may reflect atypical regulation of cortical oscillations in ASD. Prior work reports group differences in spectral power and connectivity across alpha–beta–gamma rhythms in ASD (with notable heterogeneity), supporting the idea that oscillatory dynamics can serve as informative features for classification. Proposed mechanisms include imbalances in excitatory–inhibitory (E/I) signaling and thalamocortical circuit dysfunction, which can alter the stability and amplitude of resting rhythms. Therefore, we interpret our findings as compatible with these mechanisms while acknowledging cohort size and age/sex differences. Targeted neurophysiological studies will be required to confirm causality [36].

Our study built on existing research in the field of EEG-based classification, aiming to improve ASD detection. Several recent studies related to this topic have identified advantages and drawbacks, which can be compared with the findings of this study. Compared with previous studies, which focused on various feature extraction methods and classifiers, our approach uses SWT for feature extraction and FLDA for classification. This combination offers several advantages, including effective dimensionality reduction of EEG data and accurate ASD classification. By utilizing SWT, important features can be extracted from EEG signals, thereby enabling a better representation of ASD-related brain activity patterns. However, this approach may have some drawbacks, including the use of relatively small datasets and limited FLDA capabilities. Despite these challenges, this study makes a significant contribution to the classification of ASD EEG signals with competitive accuracy while also emphasizing the efficiency of the simpler FLDA method compared to several other complex techniques used in previous studies. This research supports the development of effective and efficient automated diagnosis systems in the context of neurological disorders such as ASD. This resource-aware design supports practical adoption in low-compute clinics. Future work will explore kernel/regularized discriminants and deep learning on larger, balanced cohorts.

To situate our findings within the literature, Table 6 synthesizes representative ASD-EEG classification studies already cited in Section 1, reporting the feature/classifier pair, dataset context, and the authors’ reported accuracy. Because cohorts, acquisition protocols, and evaluation schemes differ across studies, these figures are not directly comparable; the table serves as context rather than a head-to-head benchmark. On the same KAU dataset, the Butterworth → ICA → KNN baseline reports 85.4% accuracy [9], CWT → SVM achieves 95% [12], and spectrogram/STFT pipelines reach 95.25% [22]. Under an explicit subject-wise split on KAU, our SWT (levels 3/4/6) → FLDA pipeline attains 95% accuracy in the β-band. This side-by-side view underscores the interpretability and computational efficiency of our approach alongside competitive performance.

Limitations. Our findings should be viewed in light of sex/age imbalance (ASD: five males/three females, 6–20 years; controls: eight males, 9–13 years) and a small subject count (*n* = 16). We used a subject-wise 70/30 hold-out to avoid leakage, but broader validation will benefit from sex/age-matched recruitment, subject-wise k-fold/LOSO and external testing. Analyses used 15 of 16 channels (O2 excluded), and future work will examine robust channel/reference choices and non-linear/regularized discriminants within the same evaluation protocol. A single-classifier design constrains the breadth of comparison; future work will perform subject-wise benchmarking with SVM/k-NN/RF and lightweight deep models as the cohort size and balance improve.

## 5. Conclusions

This study demonstrates that the combination of the SWT and FLDA methods significantly enhances the accuracy of EEG signal processing for both autistic and typically developing subjects, achieving an accuracy rate of over 90%. The key classification parameters included gamma signals (32–64 Hz) at level 3, representing focused brain activity, beta signals (16–32 Hz) at level 4, indicating normal brain activity, and theta signals (4–8 Hz) at level 6, corresponding to brain activity during sleep. These parameters were extracted through SWT using the Daubechies 4 (db-4) mother wavelet, along with soft thresholding for effective filtering. The system demonstrated high accuracy in classifying normal EEG signals at 96%, while the classification accuracy for autistic EEG signals reached 93%, with the slight decrease attributed to overlapping scatter in the data. These results were validated using the confusion matrix method during training and testing. The findings of this research can be applied in practical scenarios, particularly in improving the early detection and classification of autism through EEG analysis.

Additionally, this framework can be extended to more complex brain signal analyses in other neurological disorders [46,47,48], with potential wearable applications [49]. However, there are limitations, including the overlap of certain EEG patterns in autistic subjects, which slightly reduces accuracy. Future research could explore more advanced filtering techniques or a larger, more diverse dataset to address this challenge. Further exploration into more sophisticated classification algorithms may also yield higher accuracy and more robust results.

## Figures and Tables

**Table 1:** Extraction and denoising results of EEG features at level 3.

Data	FP1 (Hz)	F3 (Hz)	F7 (Hz)	T3 (Hz)	…	OZ (Hz)	Class
1	20.70515	21.71104	20.39143	6.959006	…	−10.174	Normal
2	22.19637	24.09438	22.9614	12.63111	…	−15.6247	Normal
3	24.53501	25.39331	24.71271	16.85653	…	−17.9162	Normal
…	…	…	…	…	…	…	…
8.000	25.94938	26.05881	24.2168	12.1017	…	−15.0655	Normal
8001	6.332813	−22.1256	−12.8341	7.169845	…	19.57307	ASD
8002	0.626899	−21.0605	−12.1656	3.108195	…	12.88358	ASD
8003	−0.77898	−17.5857	−17.161	3.075867	…	15.92139	ASD
…	…	…	…	…	…	…	…
16.000	14.84071	−6.8033	−7.82897	6.833994	…	16.34017	ASD

**Table 2:** Extraction and denoising results of EEG features at level 4.

Data	FP1 (Hz)	F3 (Hz)	F7 (Hz)	T3 (Hz)	…	OZ (Hz)	Class
1	20.17439	21.08247	19.67696	7.651534	…	−10.0963	Normal
2	21.38831	23.34116	21.87948	13.52822	…	−15.165	Normal
3	23.45923	24.5291	23.34335	17.9128	…	−17.1389	Normal
…	…	…	…	…	…	…	…
8000	24.60603	25.12293	22.6783	13.30784	…	−13.937	Normal
8001	6.586297	−22.0628	−12.5653	8.17759	…	19.45576	ASD
8002	1.226708	−21.3885	−11.5433	4.327005	…	13.16418	ASD
8003	0.124645	−18.2795	−16.2514	4.390527	…	16.547	ASD
…	…	…	…	…	…	…	…
16.000	16.14102	−8.07165	−6.58169	7.992751	…	17.45375	ASD

**Table 3:** Extraction and denoising results of EEG features at level 6.

Data	FP1 (Hz)	F3 (Hz)	F7 (Hz)	T3 (Hz)	…	OZ (Hz)	Class
1	20.29084	20.7321	19.22691	7.560314	…	−9.21138	Normal
2	21.54138	22.65318	21.23181	13.1909	…	−13.8999	Normal
3	23.58845	23.79831	22.53498	17.41969	…	−15.6655	Normal
…	…	…	…	…	…	…	…
8.000	24.09664	24.48455	21.55254	12.73626	…	−12.8648	Normal
8001	6.57135	−22.081	−12.5781	7.771396	…	19.67485	ASD
8002	1.582899	−21.7472	−11.0373	4.023141	…	13.54433	ASD
8003	0.970091	−18.8331	−14.6427	4.929182	…	17.00702	ASD
…	…	…	…	…	…	…	…
16.000	17.42998	−8.73002	−5.08022	9.285977	…	−18.1207	ASD

**Table 4:** Confusion matrices (counts) for ASD vs. normal by SWT level (autism = positive (+); *n* = 4800 per level).

		Prediction					
		Level 3		Level 4		Level 6	
		ASD	Normal	ASD	Normal	ASD	Normal
Actual	ASD	2208	160	2280	120	2040	350
	Normal	224	2208	120	2280	370	2040

**Table 5:** Binary classification performance by SWT level under FLDA (autism = positive).

SWT Level	Metrics				
	Accuracy	Specificity	Recall	Precision	F1 Score
Level 3	0.920	0.908	0.932	0.908	0.920
Level 4	0.950	0.950	0.950	0.950	0.950
Level 6	0.850	0.846	0.854	0.846	0.850

**Table 6:** Overview of prior ASD-EEG classification approaches.

Ref.	Method	Dataset	Result
[9]	Butterworth → ICA → KNN	KAU EEG signal (ASD)	Accuracy 85.4%
[12]	CWT features → SVM	KAU EEG signal (ASD)	Accuracy 95%
[22]	Spectrogram/STFT features → classical ML	KAU EEG signal (ASD)-related EEG task	Accuracy 95.25%
This work	SWT (Levels 3/4/6) → FLDA	KAU EEG signal (ASD) → β-band	Accuracy 95%

## Data Availability

Restrictions apply to the availability of these data. Data were obtained from King Abdulaziz University (KAU) Hospital, Jeddah, Saudi Arabia and are available from the data owner with permission (contact: Dr. Mohammed Jaffer Alhaddad, malhaddad@kau.edu.sa).

## Abbreviations

The following abbreviations are used in this manuscript:

ASD	Autism Spectrum Disorder
EEG	Electroencephalography
SWT	Stationary Wavelet Transform
DWT	Discrete Wavelet Transform
FLDA	Fisher’s Linear Discriminant Analysis
LDA	Linear Discriminant Analysis
Db4	Daubechies-4 (mother wavelet)
BCI	Brain–Computer Interface
BCI2000	Brain–Computer Interface 2000 platform (software)
STFT	Short-Time Fourier Transform
CWT	Continuous Wavelet Transform
CSP	Common Spatial Pattern
LBP	Local Binary Pattern
k-NN	k-Nearest Neighbors
SVM	Support Vector Machine
RF	Random Forest
CNN	Convolutional Neural Network
RNN	Recurrent Neural Network
LSTM	Long Short-Term Memory (network)
GRU	Gated Recurrent Unit
ANN	Artificial Neural Network
VMD	Variational Mode Decomposition
AUC	Area Under the Curve
ROC-AUC	Area Under the Receiver Operating Characteristic Curve
LOSO	Leave One Subject Out
MCI	Mild Cognitive Impairment
KAU	King Abdulaziz University
